# No correlation between amylase/trypsin-inhibitor content and amylase inhibitory activity in hexaploid and tetraploid wheat species

**DOI:** 10.1016/j.crfs.2023.100542

**Published:** 2023-06-28

**Authors:** Nora Jahn, C. Friedrich H. Longin, Katharina A. Scherf, Sabrina Geisslitz

**Affiliations:** a Department of Bioactive and Functional Food Chemistry, Institute of Applied Biosciences, Karlsruhe Institute of Technology (KIT), Adenauerring 20 a, 76131, Karlsruhe, Germany; b State Plant Breeding Institute, University of Hohenheim, 70599, Stuttgart, Germany

**Keywords:** Einkorn, Emmer, Enzyme inhibition assay, LC-MS/MS, Non-celiac wheat sensitivity (NCWS), Spelt

## Abstract

Wheat amylase/trypsin-inhibitors (ATI) are known triggers for wheat-related disorders. The aims of our study were to determine (1) the inhibitory activity against different α-amylases, (2) the content of albumins and globulins (ALGL) and total ATI and (3) to correlate these parameters in wholegrain flour of hexaploid, tetraploid and diploid wheat species.

The amount of ATI within the ALGL fraction varied from 0.8% in einkorn to 20% in spelt. ATI contents measured with reversed-phase high-performance liquid chromatography (RP-HPLC) revealed similar contents (1.2–4.2 mg/g) compared to the results determined by LC-MS/MS (0.2–5.2 mg/g) for all wheat species except einkorn. No correlation was found between ALGL content and inhibitory activity. In general, hexaploid cultivars of spelt and common wheat had the highest inhibitory activities, showing values between 897 and 3564 AIU/g against human salivary α-amylase. Tetraploid wheat species durum and emmer had lower activities (170–1461 AIU/g), although a few emmer cultivars showed similar activities at one location. In einkorn, no inhibitory activity was found. No correlation was observed between the ATI content and the inhibitory activity against the used α-amylases, highlighting that it is very important to look at the parameters separately.

## Highlights

• Content and inhibitory activity of amylase/trypsin-inhibitors (ATI) were analyzed.
• ATI contents measured with RP-HPLC correlate with those determined by LC-MS/MS.
• Inhibitory activity was higher in hexaploid than in tetraploid wheat species.
• Einkorn samples showed no inhibitory activity against human saliva α-amylase.
• No correlation was found between ATI content and inhibitory activity.

## Graphical abstract

**Figure:**
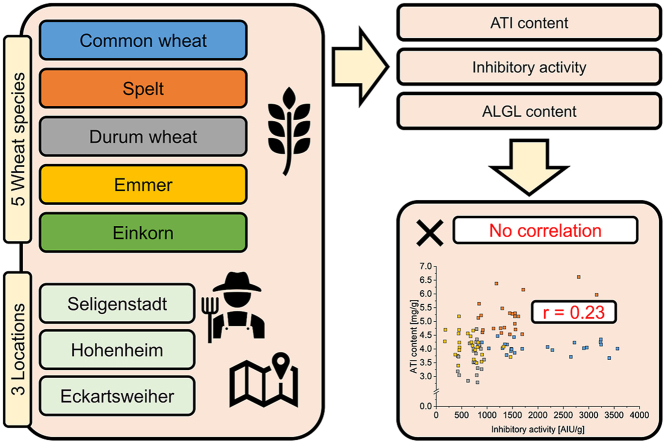

## 1 Introduction

Wheat is one of the most important staple foods worldwide. Common wheat, also known as bread wheat (*Triticum aestivum* ssp. *aestivum*), and durum wheat (*T. turgidum* ssp. *durum*) are nowadays used to produce bread and pasta and are often called modern wheat species. The “ancient” wheat species spelt (*Triticum aestivum* ssp. *spelta*), emmer (*T. turgidum* ssp. *dicoccum*) and einkorn (*T. monoccoccum*) are cultivated in very low amounts, but they are currently experiencing a rediscovery. One reason is that consumers associate a better digestibility and tolerability of ancient wheat species compared to modern ones (Teuber et al., 2016).

Wheat amylase/trypsin inhibitors (ATI) are known allergens for immunoglobulin E mediated allergies such as food allergy (Pastorello et al., 2007) and bakers’ asthma (Sander et al., 2011). Besides this, ATI trigger the innate immune system by activation of the toll-like receptor 4 (TLR4)-MD2-CD14 complex on human monocytes, macrophages and dendritic cells causing secretion of proinflammatory chemokines and cytokines (Cuccioloni et al., 2017; Junker et al., 2012; Zevallos et al., 2017). This activation may result in symptoms typical of non-celiac wheat sensitivity (NCWS) in susceptible individuals as well as in worsening of other pre-existing inflammatory reactions (Junker et al., 2012; Zevallos et al., 2018). There is a significant overlap of the symptoms of NCWS and (wheat sensitive) irritable bowel syndrome (Catassi et al., 2017), especially regarding intestinal symptoms such as stomach pain, bloating and diarrhea. In this context, two different activities of ATI have to be taken into account: (1) The activation of the TLR4 (bioactivity) and the resulting signal cascade causing intra- and extraintestinal symptoms and (2) the inhibition of the digestive enzymes α-amylase and trypsin (inhibitory activity), which can lead to intestinal problems due to incompletely digested starch and proteins as common food constituents. In addition to the bioactivity and inhibitory activity, the absolute content and thus, the presence of ATI must be considered. However, the mere presence of ATI in flour and cereal products does not necessarily confirm their activity in the human body. Currently, little is known, if the ATI content is correlated with the bioactivity and the inhibitory activity towards α-amylase and trypsin or not.

ATI, along with other inhibitors, belong to the water- and salt-soluble albumin and globulin (ALGL) fraction among wheat proteins. Purothionins, grain softness proteins and low concentrations of gliadins are also part of this fraction (DuPont et al., 2005).

ATI are divided in three groups: The first two groups contain monomeric (0.28) and dimeric (0.19 and 0.53) ATI with inhibitory activity mainly against α-amylase. The third group contains the tetrameric CM-type ATI (CM1, CM2, CM3, CM16 and CM17), which are named according to their solubility in chloroform and methanol and have activity towards both α-amylase and trypsin. The hexaploid wheat species common wheat and spelt have approximately equal proportions of monomeric, dimeric and tetrameric ATI in comparison to the tetraploid wheat species durum wheat and emmer where the CM-types are more abundant than the monomeric and dimeric ATI (Geisslitz et al., 2020; Sielaff et al., 2021). ATI correspond to 2–6% of the total protein in hexaploid and tetraploid wheat species (Geisslitz et al., 2020). The content of ATI is influenced by both the genotype and the environment and shows a high variability within each wheat species (Call, Kapeller, Grausgruber, Reiter, Schoenlechner, & D'Amico, 2020; Geisslitz et al., 2020; Prandi et al., 2013; Sielaff et al., 2021), especially in common wheat (Bose et al., 2020; Sagu et al., 2020). There are already some methods to reduce the ATI content and activity either in the grain or during food processing to enhance the tolerability of wheat. The three-dimensional structure of ATI is stabilized by four to five intramolecular disulfide bonds, which can be cleaved, e.g., using chemical reducing agents to inactivate the bioactivity (Cuccioloni et al., 2017). Sourdough fermentation using special lactobacilli can also be used to degrade ATI (Huang et al., 2020). Other alternatives are breeding and selection of cultivars with inherently low ATI content or the use of genome editing methods, which already succeeded in the reduction of ATI in common wheat and durum wheat (Camerlengo et al., 2020; Kalunke et al., 2020).

In contrast to the tetraploid and hexaploid wheat species, diploid einkorn contains very low amounts of ATI or the ATI are even not present at all, because they are not expressed (Afzal et al., 2023; Geisslitz et al., 2020; Sielaff et al., 2021; Zoccatelli et al., 2012). This is in accordance to the missing α-amylase inhibitory activity (Bedetti et al., 1974; Sanchez-Monge et al., 1996; Simonetti et al., 2022), but in contrast to a very potent inhibitory activity towards trypsin (Call et al., 2020; Simonetti et al., 2022), which might result from a specific einkorn trypsin inhibitor (Sielaff et al., 2021; Taddei et al., 2009). The inhibitory activity towards trypsin is lower in emmer than in einkorn, whereas common wheat, spelt and durum wheat are in between (Call et al., 2020). Only small differences are present in the inhibitory activity towards porcine pancreas α-amylase (PPA) and human salivary α-amylase (HSA) in emmer, durum wheat, spelt and common wheat (Gélinas and Gagnon, 2018; Simonetti et al., 2022), but the harvest year, the growing location, the genotype and the interaction between these factors influence the inhibitory activity against HSA (Simonetti et al., 2022).

The correlation between the inhibitory activity and the content of ATI and ALGL in wheat and related wheat species has not yet been evaluated. Therefore, the aim of this study was to analyze the inhibitory activity against PPA and HSA in different cultivars of common wheat, spelt, durum wheat, emmer and einkorn to answer the question whether ATI inhibitory activity and content of ATI and ALGL are correlated or not. A second aim was to determine whether reversed-phase high-performance liquid chromatography (RP-HPLC) analysis of the ALGL fraction could be used to estimate the ATI content.

## 2 Materials and methods

### 2.1 Materials

Eight cultivars each of common wheat, spelt, durum wheat, emmer and einkorn were cultivated by the State Plant Breeding Institute, University of Hohenheim (Stuttgart, Germany) at three locations in Germany (Seligenstadt, Hohenheim and Eckartsweiher), and harvested in 2013. The kernels were milled with an ultra centrifugal mill ZM 200 (Retsch, Haan, Germany) with a 0.5 mm sieve before analysis and stored for at least two weeks.

The agronomic performance (Longin et al., 2015), the gluten protein composition (Geisslitz et al., 2019) and the quantitation of ATI concentrations (Geisslitz et al., 2020) of the sample set have already been described in detail.

### 2.2 Quantitation of albumins and globulins

ALGL were extracted according to the modified Osborne fractionation (Wieser et al., 1998). In short, flour (100 mg) was extracted two times with a salt solution (2 × 1.0 mL, 0.4 mol/L NaCl with 0.067 mol/L Na_2_HPO_4_/KH_2_PO_4_, pH 7.6). Each extraction step started with vortex mixing for 2 min at 22 °C followed by magnetic stirring for 10 min at 22 °C. After centrifugation for 25 min at 22 °C and 3750×*g* the supernatants were combined and filled up to 2 mL with salt solution. The supernatants were filtered (Whatman^TM^, AQUA30/0.45 CA, GE Healthcare, Freiburg, Germany) and analyzed by RP-HPLC. All extractions were made in triplicate.

For RP-HPLC analysis, a Prominence UFLC (Shimadzu, Kyoto, Japan) equipped with a YMC-Triart Bio C_18_ (3 μm, 2.1 × 150 mm) was used. Solvent A was water containing 0.1% trifluoroacetic acid and solvent B was ACN containing 0.1% trifluoroacetic acid. The flow rate was 0.5 mL/min, the column was tempered at 60 °C and following gradient was applied: 0–0.4 min 0% B, 0.4–0.5 min 0–20% B, 0.5–8.0 min 20–60% B, 8.0–8.1 min 60–90% B, 8.1–13.0 min 90% B, 13.0–13.1 min 90–0% B, 13.1 min–27 min 0% B. Detection was done by measuring the UV absorbance at 210 nm. Control of the system and integration were performed with LabSolutions (Shimadzu). For external calibration and calculation of the ALGL content, reference gliadin (2.5 mg/mL) from the Prolamin Working Group (PWG-gliadin) (van Eckert et al., 2006) was dissolved in 60% ethanol (v/v), ultrasonicated and filtered. Different volumes (5, 10, 15, and 20 μL) were analyzed.

**Fig. 1:**
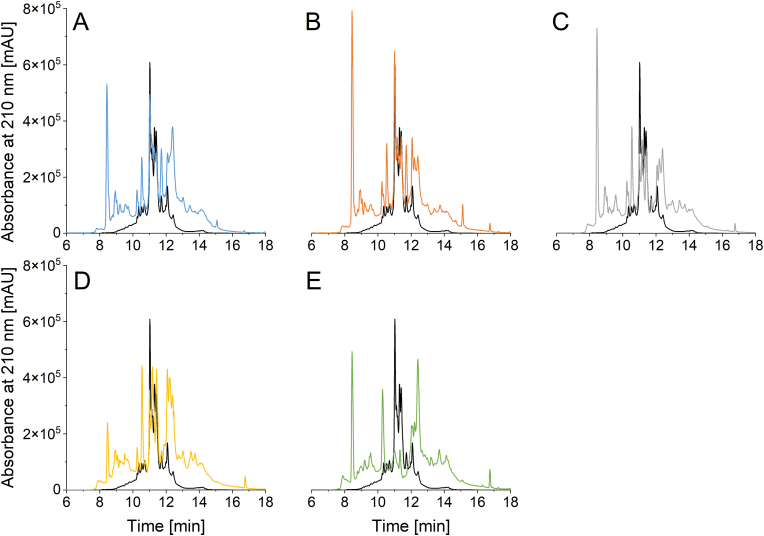
Reversed-phase high-performance liquid chromatography profiles of the commercially available α-amylase inhibitor from *T. aestivum* (black) and albumin and globulin fractions of the cultivars (A) Mulan (common wheat), (B) Badenkrone (spelt), (C) Wintergold (durum wheat), (D) Ramses (emmer), and (E) Tifi (einkorn).

For the identification of α-amylase inhibitors within the RP-HPLC chromatograms of the ALGL fraction, a commercially available α-amylase inhibitor from *T. aestivum* (2.5 mg/mL) (A1520; Sigma-Aldrich) was dissolved in salt solution. Volumes of 2, 5, 10 and 15 μL were injected to determine the specific retention time window of ATI within the ALGL fraction and to obtain an external calibration to estimate the ATI content. A purity of 19% (Geisslitz et al., 2018) was used for the calculation. The RP-HPLC chromatograms of ALGL (Fig. 1) from all samples were integrated from 10.9 to 11.9 min to calculate the ATI content, designated as ATI (HPLC) in the following.

### 2.3 Inhibitory activity against human and porcine α-amylase

The inhibitory activity towards PPA (A3176; Sigma-Aldrich, Oakville, Canada) and HSA (A1031; Sigma-Aldrich) was determined with the EnzChek^TM^ Ultra Amylase Assay Kit (Thermo Fisher Scientific, Waltham, MA, USA) according to Gélinas and Gagnon (2018) with small modifications. Flour (100 mg) was extracted with extraction buffer (1 mL, 20 mmol/L Na_2_HPO_4_ × 2 H_2_O, 100 mmol/L NaCl, pH 7.5) for 1 h at 22 °C. After centrifugation for 25 min at 22 °C and 3750×*g*, the supernatant was incubated for 20 min at 80 °C under shaking, to inactivate endogenous enzymes. The suspension was again centrifuged and the resulting supernatant was diluted to obtain a slope that is ideally half as high as the slope of the positive sample (25 μL extraction buffer and 25 μL α-amylase solution) (see formula 1). All extractions were made in triplicate.

The DQ™-starch reagent was dissolved in sodium acetate buffer (100 μL, 50 mmol/L) and diluted with extraction buffer (900 μL). The reagent was stored in the dark. Directly before use, the reagent was again diluted 1:10 with extraction buffer.

α-Amylase solutions were prepared fresh every day. α-Amylase of different origins (1 mg) was dissolved in 1 mL of water (HSA) or extraction buffer (PPA) and diluted with extraction buffer to obtain linear formation of the fluorescent product.

Fluorescence was measured with a multiplate reader (Tecan, Maennedorf, Switzerland) at an excitation wavelength of 485 nm and an emission wavelength of 515 nm as a continuous determination over 15 min in intervals of 20 s. The inhibitory activity was calculated as the comparison of the positive sample and the sample (25 μL diluted flour extract and 25 μL α-amylase solution). Both were corrected with blanks containing extraction buffer instead of α-amylase solution. The solutions were pipetted in 96-well plates and incubated for 10 min. Then, 50 μL reagent solution was added and the measurement was started immediately.

The slopes (m) of the linear product formation were calculated and considered in formula 1:

$$Inhibitory\;activity\;(AIU/g\;flour)=\frac{\left(\frac{m_{Positive}-m_{Sample}}{m_{Positive}}\right)\times Dilution\;factor}{Sample\;weight}$$

m = Slope for linear product formation
Dilution factor = Common wheat: 100–500; spelt: 50–500; durum wheat: 10–100; emmer: 10–500; einkorn: 1 (undiluted)

### 2.4 Statistical analyses

Pearson correlation coefficients and one-way and two-way analysis of variance (ANOVA) with Tukey’s test (p < 0.05) were calculated with Origin 2021 (OriginLab, Northampton, Massachusetts, USA). The following thresholds for the coefficient of correlation (r) were defined: ±0.54 < r ≤ ±0.67: weak correlation; ±0.67 < r ≤ ±0.78: medium correlation; ±0.78 < r ≤ ±1.00: strong correlation (Thanhaeuser et al., 2014). Einkorn samples had no activity against both α-amylases and very low ATI contents. Therefore, the correlation analyses were performed considering only the cultivars of common wheat, spelt, durum wheat and emmer, unless indicated otherwise.

## 3 Results and discussion

### 3.1 Content of albumins and globulins

**Fig. 2:**
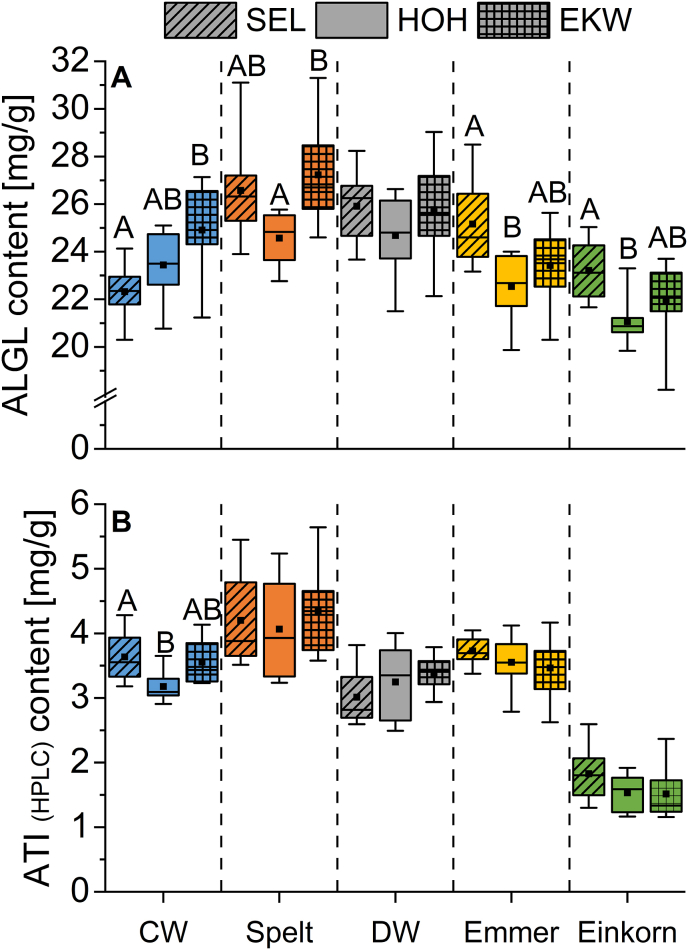
Contents of albumins and globulins (ALGL) (A) and amylase/trypsin-inhibitors (ATI) (B) measured with reversed-phase high-performance liquid chromatography (RP-HPLC) in common wheat (CW), spelt, durum wheat (DW), emmer and einkorn. Data of eight cultivars per wheat species and growing location (SEL, Seligenstadt; HOH, Hohenheim; EKW, Eckartsweiher) are presented. The boxes correspond to 25th and 75th percentiles. The square in the box indicates the mean value, the line the median. The whiskers designate minima and maxima. Different capital letters indicate significant differences between growing locations within each wheat species (one-way ANOVA, Tukey's test at p < 0.05).

In general, spelt and durum wheat had the highest mean ALGL content (26.1 and 25.4 mg/g), followed by emmer (23.7 mg/g) and common wheat (23.6 mg/g) (Fig. 2A, Supplementary Table 1). Einkorn showed the lowest ALGL content (22.1 mg/g). Regarding the different growing locations, all wheat species except common wheat had the lowest ALGL content at Hohenheim. For common wheat, the lowest content was found at Seligenstadt (22.3 mg/g), whereas Eckartsweiher showed the highest one (24.9 mg/g). Durum wheat, emmer and einkorn had the highest ALGL content at Seligenstadt, but spelt had the highest ALGL content at Eckartsweiher, similar to common wheat. A two-way ANOVA revealed that the wheat species (F = 22.9; p < 0.05) had a higher influence on the ALGL content than the growing location (F = 8.9; p < 0.05). This underlines the necessity to investigate samples of different varieties per species, which have been grown at the same but diverse set of environments to elaborate general comparisons of different crops. There were no cultivars that were characterized by a particularly high or low content at all locations. Considering the total ATI content determined by liquid chromatography-tandem mass spectrometry (LC-MS/MS) as sum of 13 ATI reported in Geisslitz et al. (2020), the mean percentage of ATI based on the ALGL content was 17.4% for common wheat, 20.0% for spelt, 14.7% for durum wheat, 17.5% for emmer and 0.8% for einkorn. A weak correlation between the ALGL and the ATI content (LC-MS/MS) was observed (r = 0.48).

### 3.2 Content of amylase/trypsin-inhibitors measured with high-performance liquid chromatography

The RP-HPLC peak profiles of one cultivar of each wheat species compared to the α-amylase inhibitor from *T. aestivum* are shown in Fig. 1. The ALGL of the different cultivars were eluted between 8 and 16 min, whereas the inhibitor standard was eluted between 9 and 13 min with three very dominant peaks between 10.9 and 11.6 min. All wheat species except einkorn showed clear peaks in that timeframe.

Integration of the peaks from 10.9 to 11.9 min and calibration via the α-amylase inhibitor resulted in the content of ATI (HPLC). Spelt samples had the highest mean content (4.2 mg/g), followed by emmer (3.6 mg/g), common wheat (3.4 mg/g) and durum wheat (3.2 mg/g). Einkorn samples had contents between 1.2 and 2.6 mg/g (Fig. 2B; Supplementary Table 2). ATI (HPLC) contents for all wheat species except einkorn were on average as high as those determined by LC-MS/MS (Geisslitz et al., 2020). Using LC-MS/MS, spelt also had the highest ATI content (5.2 mg/g), followed by emmer and common wheat (4.1 mg/g, respectively) and durum wheat (3.7 mg/g). Einkorn had almost no ATI (0.2 mg/g). The results are also comparable to those obtained by Call et al. (2020), although their mean values were higher in general. This may be due to the different reference standard used for quantitation (trypsin inhibitor from soybean was used instead of α-amylase inhibitor from wheat) and the different set of samples.

**Fig. 3:**
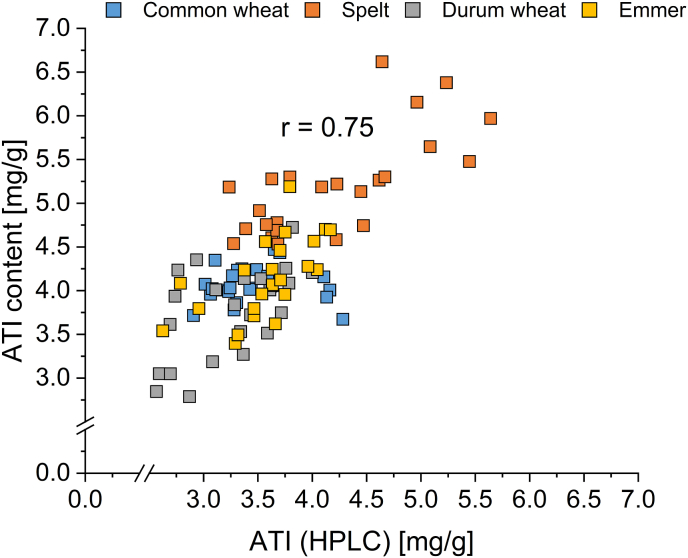
Correlation diagram between amylase/trypsin-inhibitors (ATI) measured with high-performance liquid chromatography (HPLC) and total ATI content determined by LC-MS/MS (Geisslitz et al., 2020).

A correlation coefficient of r = 0.75 was obtained, when correlating the ATI (HPLC) content and the total ATI content measured with LC-MS/MS (Fig. 3). There was a strong positive correlation (r = 0.91) when considering all cultivars from all five wheat species, i.e., including einkorn (correlation not shown).

However, the method is not suitable for a reliable quantitation of ATI. Other proteins, especially enzymes and enzyme-inhibitors could be present in the integrated time interval of the samples since the ALGL extract was used. Call et al. (2019) showed that saline extracts of wheat contain proteins, such as grain softness protein, non-specific lipid transfer proteins and β-amylase. Due to similar molecular masses, it is more than presumably that these proteins elute in the interval of interest. Thus, the ATI content could be overestimated in our study.

The α-amylase inhibitor might not be the ideal standard due to its low purity of about 19%, also containing other proteins in the integrated time interval and thus distort the actual ATI content. In addition, not all ATI are present in the standard (Geisslitz et al., 2018).

Furthermore, it is not possible to quantify single ATI with RP-HPLC as it is with the LC-MS/MS method. Although the total ATI contents measured with this method are comparable with the values of the ATI (HPLC) for common wheat, spelt, durum wheat and emmer, greater discrepancies result for the einkorn samples, because the ATI (HPLC) values are up to 10-fold higher than the ones measured with LC-MS/MS. However, this could also be an advantage, since einkorn presumably contains more ATI than are currently detected by the LC-MS/MS method. For example, Sielaff et al. (2021) found clear evidence for an einkorn-specific inhibitor, of which no peptides have been included in the LC-MS/MS method so far. There are also some more advantages. The method is comparatively easy to perform and requires less time compared to the LC-MS/MS method. It also uses fewer reagents and more commonly accessible laboratory equipment.

These results indicate that a quantitation via the inhibitor standard provides a quick and easy estimate of the total ATI content. To enable precise quantitation of single ATI it is essential that more specific analytical methods like the LC-MS/MS method are used.

### 3.3 Inhibitory activity against porcine pancreas α-amylase

**Fig. 4:**
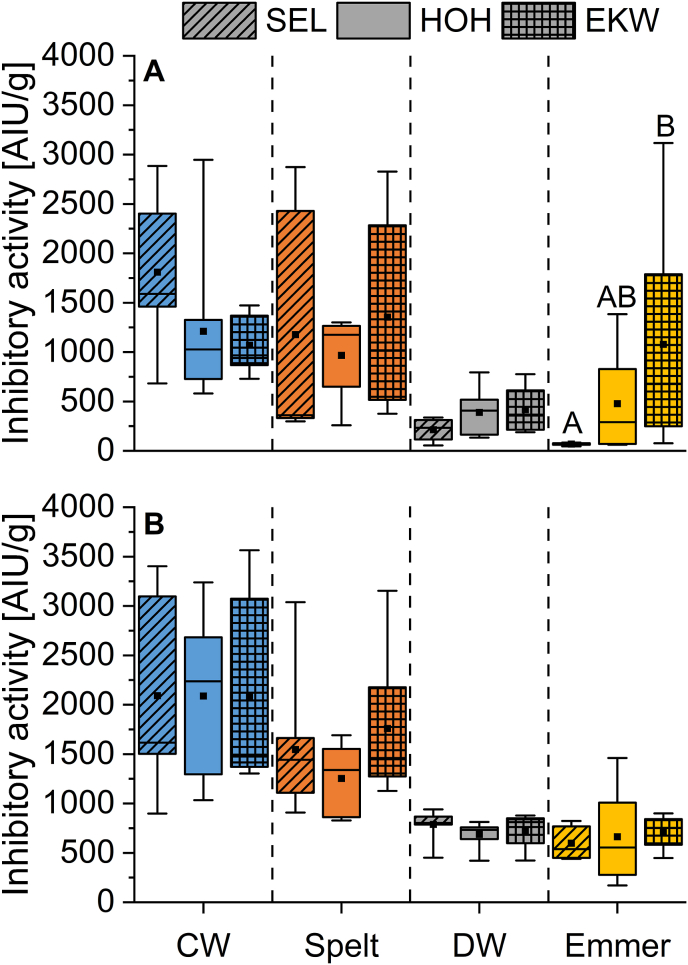
Inhibitory activity against porcine pancreas amylase (A) and human salivary α-amylase (B) in common wheat (CW), spelt, durum wheat (DW) and emmer. No data of einkorn is shown, because none of the cultivars had a detectable inhibitory activity against the amylases. Data are shown as in Fig. 2. Different capital letters indicate significant differences between growing locations within each wheat species (one-way ANOVA, Tukey's test at p < 0.05).

Over all growing locations, common wheat and spelt had the highest activities against the PPA, even if a few emmer cultivars grown in Eckartsweiher had similar inhibitory activities. No inhibitory activity against PPA was found in all einkorn cultivars of different growing locations. Common wheat showed inhibitory activities between 637 and 2947 AIU/g, having the highest mean activity in Seligenstadt (1809 AIU/g). The inhibitory activity of spelt ranged from 299 to 2859 AIU/g (Fig. 4A, Supplementary Table 3).

The tetraploid wheat cultivars durum wheat and emmer had lower inhibitory activities compared to the hexaploid ones. Durum wheat showed similar activities at all locations, ranging from 56 AIU/g in Seligenstadt up to 776 AIU/g in Eckartsweiher. The inhibitory activities of the emmer cultivars were between 44 and 3117 AIU/g, showing the greatest range of all locations and cultivars.

Over all wheat species and growing locations, emmer cultivars from Seligenstadt had the lowest mean inhibitory activity against PPA (69 AIU/g).

One-way ANOVA revealed that there were only significant differences for the emmer cultivars grown in Seligenstadt (lowest mean value) and Eckartsweiher (highest mean value with 1078 AIU/g). Two-way ANOVA indicated that the wheat species (F = 12.6; p < 0.05) had a larger influence on the activity than the growing location (F = 0.9; p < 0.05).

### 3.4 Inhibitory activity against human saliva α-amylase

The inhibitory activities of the different wheat species against the HSA were mostly similar to the PPA (Fig. 4B, Supplementary Table 4). Einkorn also showed no activity against HSA. This is in accordance with the results by Simonetti et al. (2022). Furthermore, these findings match those reported in Geisslitz et al. (2020) and in Afzal et al. (2023), because einkorn cultivars only contained few single ATI and in lower quantities compared to common wheat as analyzed by LC-MS/MS. This emphasizes that clinical studies with well-characterized and representative einkorn samples are required to understand the role of einkorn ATI in context of wheat-related disorders more in detail.

The hexaploid wheat species common wheat (897–3564 AIU/g) and spelt (908–3154 AIU/g) had the highest activities. The tetraploid species had lower activities between 421 and 940 AIU/g and 170–1461 AIU/g for durum wheat and emmer, respectively. Similar to the PPA, the tetraploid wheat species had a lower inhibitory activity than the hexaploid ones.

One-way ANOVA revealed no significant differences between different locations within one wheat species. Two-way ANOVA indicated that the wheat species (F = 33.0; p < 0.05) had a more pronounced influence on the activity than the growing location (F = 0.5; p < 0.05).

In general, it was not possible to differentiate between ancient and modern species based on the inhibitory activity. These findings are supported by other studies (Gélinas and Gagnon, 2018; Simonetti et al., 2022).

### 3.5 Comparison of inhibitory activity against both α-amylases

Two different α-amylases were used for the assay. HSA was chosen because it most closely resembles human digestion. Since both α-amylases are similar in terms of molecular weight and overall topology (Kaur et al., 2014), PPA was also tested, because it is much cheaper than HSA.

The mean inhibitory activities of the wheat species were mostly comparable between the two α-amylases with the exception of the aforementioned emmer cultivars grown in Eckartsweiher. Higher inhibitory activities were generally detected against HSA than PPA, confirming the findings by Gélinas and Gagnon (2018). They also reported that most of the hexaploid wheat cultivars had a higher activity than the tetraploids. Nevertheless, they found a poor correlation (r = 0.45) between the results using the two α-amylases. The correlation between the inhibitory activity of PPA and HSA in our study was also weak (r = 0.59).

This could be explained by the different structure of the two amylases. While both PPA and HSA have a total number of 496 amino acids and the same amino acids at the catalytic site, they differ slightly in molecular weight and residues at sites that are important for substrate binding, resulting in different specificities towards substrates (Kaur et al., 2014).

In general, the assay for the measurement of the inhibitory activity involves simple sample preparation, is relatively fast and requires little instrumental effort. Nevertheless, the inhibitory activity showed high CVs (up to 25%) compared to the methods for measuring the ATI and the ALGL content (about 5%). Only poor reproducibility was achieved requiring several repetitions for some measurements. This could be explained by the enzymatic reaction in the assay, because enzymes are very sensitive to small variations in temperature, solvent composition and pH.

Furthermore, other studies use different enzymatic assays and apply different calculation methods. For example, Gélinas and Gagnon (2018) used the same assay kit as we did, but calculated the half-maximal inhibitory concentration (IC_50_) based on acarbose as external standard. Simonetti et al. (2022) used a different amylase assay kit which is based on the production of a colorimetric product at 405 nm (*p*-nitrophenol). The inhibitory activity was indicated as a percentage with respect to the control sample. Tchewonpi Sagu et al. (2019) used a method based on the use of 2,3 dinitrosalicylic acid reagent and measurement of absorption at 540 nm. They calculated the inhibition of amylase activity with the same formula as Simonetti et al. (2022). Since both groups used different methods, it was not possible to adopt the calculation. This unfortunately leads to limited comparability between the different studies.

### 3.6 Correlations of inhibitory activity, albumin and globulin and amylase/trypsin inhibitor content

**Fig. 5:**
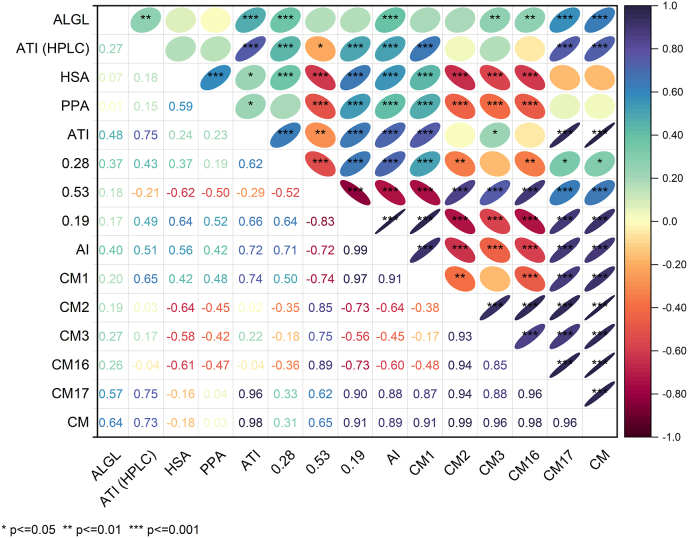
Correlation matrix showing Pearson correlation coefficients between the albumin and globulin (ALGL) content, amylase/trypsin-inhibitor (ATI) content measured with high-performance liquid chromatography (HPLC), the inhibitory activity towards human salivary α-amylase (HSA) and porcine pancreas α-amylase (PPA), total amylase/trypsin inhibitor (ATI) content, the sum of the amylase inhibitors 0.28, 0.53 and 0.19 (AI) and the sum of all chloroform/methanol (CM) soluble types.

A correlation matrix showing Pearson correlation coefficients between the inhibitory activity towards PPA and HSA and the content of ALGL, ALGL (HPLC) and different ATI is displayed in Fig. 5. All samples were considered except einkorn, because einkorn had no detectable inhibitory activity against PPA or HSA. There was no correlation between the inhibitory activity against PPA or HSA and the ALGL content of the different wheat species (r = 0.01 and r = 0.07, respectively). The lack of correlation between ALGL content and inhibitory activity can be explained by the fact that other enzymes and enzyme inhibitors are also present in the ALGL fraction (Altenbach et al., 2020; Victorio et al., 2018) and the ATI corresponded only to a maximum of 25% of the ALGL content.

Correlation analysis was also performed between the inhibitory activity and the ATI contents reported in our earlier study (Geisslitz et al., 2020). There was no correlation between the total ATI content and the inhibitory activity against PPA (r = 0.23) and HSA (r = 0.24). Furthermore, there was no correlation between the content of ATI (HPLC) and the inhibitory activity against both α-amylases (r = 0.15 and r = 0.18, respectively).

In general, weak correlations were only found between the content of single ATI and the HSA. No correlations were found at all for the PPA. There were also no correlations between the different parameters within the individual wheat species.

The 0.19 dimeric ATI showed a weak positive correlation with HSA (r = 0.64). Regarding the sum of the α-amylase inhibitors ATI 0.19, 0.28 and 0.53, almost the same result was obtained (r = 0.56). We revealed in our previous study (Geisslitz et al., 2020) that durum wheat and emmer contained lower amounts of ATI 0.19, but higher amounts of ATI 0.53 than common wheat and spelt. Furthermore, only some durum wheat cultivars contained ATI 0.28. Only weak positive correlations were observed between the activity against HSA and the content of ATI 0.19 (r = 0.64) and the content of all α-amylase inhibitors (r = 0.56). With ATI 0.53 showing a weak negative correlation (r = −0.62) and ATI 0.28 showing no correlation (r = 0.37), it might be concluded that ATI 0.19 is most dominant in the group of α-amylase inhibitors. The statistical analysis proved the weak correlation between the inhibitory activity against HSA and ATI 0.19, but this correlation was less pronounced than expected. In combination with the missing correlation to ATI 0.28 and 0.53, this shows the high complexity and a possible interaction between HSA and various ATI.

Next to 0.19, the tetrameric CM3 is the most bioactive ATI in wheat (Zevallos et al., 2017). The content of CM3 and the activity against HSA showed a weak negative correlation (r = −0.58). No correlation was observed between both α-amylases and the sum of all CM-types (CM1, CM2, CM3, CM16 and CM17). In addition, only weak negative correlations were found between the activity against HSA and the content of ATI CM2 (r = −0.64) and CM16 (r = −0.61). The negative correlation between the inhibitory activity and single CM-types might indicate that the CM-types indeed play a minor role in the inhibition of α-amylase. Alternatively, it might be possible that the assay is more sensitive to the monomeric and dimeric ATI (α-amylase inhibitors) than to the group of tetrameric ATI (CM-types). To prove this, either standard solutions of the single ATI purified from wheat flour or recombinant ATI proteins need to be tested with the enzymatic assay.

## 4 Conclusion

The aim of this study was to investigate the correlation between inhibitory activity against α-amylases from different sources (PPA and HSA) and the ATI and ALGL content in tetraploid and hexaploid wheat species. The hexaploid wheat cultivars of common wheat and spelt had higher inhibitory activities against PPA and HSA than the tetraploid species durum wheat and emmer. Diploid einkorn had no inhibitory activity against HSA or PPA. A weak positive correlation was found between the results using these two α-amylases. A correlation was found between the content of ATI (HPLC) and the total ATI content measured with LC-MS/MS. However, there were no correlations between the total ATI content, the inhibitory activity and the ALGL content, respectively. In addition, there were only weak or even no correlations between the contents of the individual ATI and the inhibitory activity against the two α-amylases.

Reliable assays to predict the immunoreactivity of flour and its products, such as this assay for the determination of the inhibitory activity, are of great importance to people affected by wheat-related disorders. Since the different parameters are not correlated it is necessary, that there are separate analytical methods for the ALGL content, the inhibitory activity and the ATI content. Future research may also include the bioactivity of the ATI and the inhibitory activity against trypsin.

## Funding

This IGF project of the 10.13039/501100008465FEI was supported via AiF within the program for promoting the Industrial Collective Research (IGF) of the German Ministry of Economics and Climate Action (BMWK), based on a resolution of the German Parliament. Projects AiF 18355 N and AiF 19924 N.

## CRediT authorship contribution statement

**Nora Jahn:** Data curation, Formal analysis, Investigation, Methodology, Visualization, Writing – original draft, Writing – review & editing. **C. Friedrich H. Longin:** Conceptualization, Funding acquisition, Project administration, Resources, Writing – review & editing. **Katharina A. Scherf:** Conceptualization, Funding acquisition, Project administration, Resources, Supervision, Writing – review & editing. **Sabrina Geisslitz:** Conceptualization, Data curation, Funding acquisition, Investigation, Project administration, Supervision, Writing – original draft, Writing – review & editing.

## Declaration of competing interest

The authors declare that they have no known competing financial interests or personal relationships that could have appeared to influence the work reported in this paper.

## Data Availability

The LC-MS/MS data have been already deposited to the ProteomeXchange Consortium (PXD020714). The detailed ALGL and ATI (HPLC) content and the inhibitory activity are summarized in the Supplement.

## Abbreviations

ATI: amylase/trypsin inhibitor
TLR4: toll-like receptor 4
NCWS: non-celiac wheat sensitivity
ALGL: albumin and globulin
PPA: porcine pancreas α-amylase
HAS: human salivary α-amylase
RP-HPLC: reversed-phase high-performance liquid chromatography
PWG-gliadin: Prolamin Working Group
ANOVA: analysis of variance
LC-MS/MS: liquid chromatography-tandem mass spectrometry
