# Supplementary material for: No correlation between amylase/trypsin-inhibitor content and amylase inhibitory activity in hexaploid and tetraploid wheat species
Source: Curr Res Food Sci. 2023 Jun 28;7:100542. doi: 10.1016/j.crfs.2023.100542 (PMC10728332; doi:10.1016/j.crfs.2023.100542)
Supplement: Multimedia component 1 [file mmc1.docx]

Table S1. Albumin and globulin contents [mg/g] of the different wheat species and growing locations.

| Wheat species | Location | | | | | |
| --- | --- | --- | --- | --- | --- | --- |
| Common wheat | **Seligenstadt** | **±** | **Hohenheim** | **±** | **Eckartsweiher** | **±** |
| Event | 24.1 | 0.23 | 24.7 | 0.76 | 24.3 | 0.91 |
| Genius | 22.0 | 0.75 | 24.8 | 0.62 | 26.2 | 0.44 |
| JB Asano | 21.6 | 0.17 | 20.8 | 0.06 | 21.2 | 0.64 |
| Lear | 22.0 | 0.45 | 24.3 | 0.55 | 24.4 | 0.15 |
| Mulan | 22.7 | 0.31 | 25.1 | 0.40 | 26.9 | 0.17 |
| Tabasco | 23.0 | 0.59 | 22.6 | 0.25 | 24.8 | 0.06 |
| Tobak | 22.9 | 0.35 | 22.7 | 0.32 | 27.1 | 0.29 |
| Tommi | 20.3 | 0.69 | 22.6 | 0.10 | 24.3 | 0.21 |
| Spelt | | | | | | |
| Badengold | 23.9 | 0.20 | 23.1 | 0.60 | 24.6 | 0.20 |
| Badenkrone | 26.6 | 0.55 | 22.8 | 0.85 | 25.3 | 0.65 |
| Badenstern | 25.8 | 0.53 | 25.4 | 0.75 | 27.1 | 0.38 |
| Filderstolz | 27.8 | 1.31 | 24.4 | 0.29 | 27.4 | 0.32 |
| Franckenkorn | 31.1 | 0.36 | 25.6 | 0.49 | 31.3 | 0.44 |
| Oberkulmer Rotkorn | 26.1 | 0.57 | 25.8 | 0.32 | 29.5 | 0.61 |
| Schwabenkorn | 26.5 | 0.66 | 25.3 | 0.80 | 26.3 | 0.81 |
| Zollernspelz | 24.8 | 0.10 | 24.2 | 0.58 | 26.3 | 0.35 |
| Durum Wheat | | | | | | |
| Auradur | 26.9 | 0.17 | 25.8 | 0.76 | 27.5 | 0.81 |
| W-05005/02 | 28.2 | 0.74 | 26.5 | 0.80 | 29.0 | 0.91 |
| Elsadur | 26.5 | 1.01 | 26.6 | 0.93 | 26.9 | 1.47 |
| Karur | 24.3 | 0.31 | 22.7 | 0.67 | 25.6 | 0.31 |
| Logidur | 23.7 | 0.78 | 21.5 | 0.53 | 22.1 | 0.32 |
| Lunadur | 25.1 | 0.15 | 24.8 | 0.40 | 25.6 | 0.90 |
| Lupidur | 26.6 | 0.86 | 24.8 | 0.67 | 25.2 | 0.35 |
| Wintergold | 26.0 | 0.96 | 24.8 | 0.67 | 24.1 | 0.00 |
| Emmer | | | | | | |
| CC1E-04058/01 | 23.8 | 0.93 | 24.0 | 0.40 | 25.6 | 0.31 |
| CC1E-04059/01 | 23.2 | 0.38 | 19.9 | 0.55 | 23.5 | 0.21 |
| E-07087/02 | 28.5 | 0.52 | 22.4 | 0.17 | 24.3 | 0.25 |
| 9.105/06/01 | 25.2 | 1.39 | 23.6 | 0.47 | 20.3 | 0.44 |
| 9.121/05 Heuholzer Kolben | 24.5 | 1.22 | 23.0 | 0.40 | 24.7 | 0.00 |
| Osiris | 24.7 | 0.56 | 22.4 | 0.29 | 23.8 | 0.15 |
| Ramses | 27.7 | 1.17 | 24.0 | 0.20 | 23.2 | 0.59 |
| 9.131/05 Teutonia | 23.7 | 0.49 | 21.1 | 0.95 | 21.8 | 0.40 |
| Einkorn | | | | | | |
| M-04018/01 | 24.3 | 0.67 | 20.9 | 0.40 | 23.5 | 0.21 |
| 8.108/04 | 24.3 | 0.40 | 21.5 | 0.53 | 22.3 | 0.40 |
| M-04033/03 | 21.7 | 0.47 | 20.8 | 0.49 | 23.7 | 1.08 |
| M-07006/01 | 21.7 | 0.30 | 20.6 | 0.58 | 21.8 | 0.90 |
| Monlis | 22.5 | 0.47 | 20.7 | 0.64 | 21.8 | 0.46 |
| MVMenket | 23.5 | 0.47 | 23.3 | 0.44 | 22.7 | 0.53 |
| Terzino | 25.0 | 0.74 | 20.9 | 0.35 | 21.2 | 0.51 |
| Tifi | 22.8 | 0.60 | 19.8 | 0.67 | 18.2 | 0.30 |

Table S2. Amylase/trypsin-inhibitors contents [mg/g] of the different wheat species and growing locations measured with reversed-phase high-performance liquid chromatography.

| Wheat species | Location | | | | | |
| --- | --- | --- | --- | --- | --- | --- |
| Common wheat | **Seligenstadt** | **±** | **Hohenheim** | **±** | **Eckartsweiher** | **±** |
| Event | 3.70 | 0.09 | 3.65 | 0.08 | 3.59 | 0.05 |
| Genius | 4.16 | 0.03 | 3.07 | 0.06 | 4.13 | 0.09 |
| JB Asano | 3.31 | 0.10 | 2.91 | 0.02 | 4.11 | 0.01 |
| Lear | 3.18 | 0.05 | 3.11 | 0.01 | 3.23 | 0.07 |
| Mulan | 3.62 | 0.08 | 3.31 | 0.01 | 3.44 | 0.06 |
| Tabasco | 3.35 | 0.03 | 3.28 | 0.01 | 3.27 | 0.07 |
| Tobak | 4.28 | 0.05 | 3.01 | 0.02 | 3.43 | 0.09 |
| Tommi | 3.49 | 0.06 | 3.08 | 0.01 | 3.24 | 0.08 |
| Spelt | | | | | | |
| Badengold | 3.51 | 0.04 | 3.24 | 0.05 | 4.47 | 0.02 |
| Badenkrone | 3.68 | 0.06 | 4.23 | 0.07 | 4.22 | 0.03 |
| Badenstern | 3.63 | 0.02 | 3.39 | 0.07 | 3.58 | 0.08 |
| Filderstolz | 3.67 | 0.07 | 3.28 | 0.04 | 3.69 | 0.07 |
| Franckenkorn | 4.96 | 0.10 | 5.24 | 0.08 | 4.64 | 0.04 |
| Oberkulmer Rotkorn | 5.45 | 0.06 | 8.09 | 0.16 | 5.64 | 0.10 |
| Schwabenkorn | 4.09 | 0.09 | 3.63 | 0.02 | 3.80 | 0.05 |
| Zollernspelz | 4.62 | 0.08 | 4.45 | 0.05 | 4.67 | 0.10 |
| Durum wheat | | | | | | |
| Auradur | 2.77 | 0.07 | 3.76 | 0.01 | 3.79 | 0.14 |
| W-05005/02 | 3.82 | 0.07 | 2.74 | 0.07 | 2.94 | 0.04 |
| Elsadur | 3.29 | 0.06 | 3.11 | 0.07 | 3.38 | 0.11 |
| Karur | 2.87 | 0.08 | 2.57 | 0.05 | 3.08 | 0.11 |
| Logidur | 2.59 | 0.08 | 3.72 | 0.06 | 3.43 | 0.04 |
| Lunadur | 2.70 | 0.01 | 2.49 | 0.06 | 3.34 | 0.08 |
| Lupidur | 3.37 | 0.06 | 3.59 | 0.01 | 3.61 | 0.15 |
| Wintergold | 2.69 | 0.10 | 4.00 | 0.01 | 3.53 | 0.07 |
| Emmer | | | | | | |
| CC1E-04058/01 | 3.38 | 0.07 | 2.79 | 0.06 | 2.96 | 0.04 |
| CC1E-04059/01 | 3.63 | 0.03 | 3.46 | 0.08 | 2.63 | 0.03 |
| E-07087/02 | 3.79 | 0.06 | 4.12 | 0.09 | 4.17 | 0.09 |
| 9.105/06/01 | 4.02 | 0.02 | 3.70 | 0.02 | 3.54 | 0.02 |
| 9.121/05 Heuholzer Kolben | 3.75 | 0.03 | 3.63 | 0.15 | 3.71 | 0.01 |
| Osiris | 4.05 | 0.08 | 3.46 | 0.03 | 3.66 | 0.04 |
| Ramses | 3.57 | 0.10 | 3.96 | 0.03 | 3.75 | 0.10 |
| 9.131/05 Teutonia | 3.63 | 0.06 | 3.29 | 0.10 | 3.32 | 0.10 |
| Einkorn | | | | | | |
| M-04018/01 | 2.08 | 0.08 | 1.80 | 0.03 | 1.26 | 0.01 |
| 8.108/04 | 1.46 | 0.01 | 1.25 | 0.05 | 1.30 | 0.02 |
| M-04033/03 | 2.06 | 0.08 | 1.92 | 0.03 | 1.37 | 0.05 |
| M-07006/01 | 1.30 | 0.01 | 1.17 | 0.03 | 1.21 | 0.03 |
| Monlis | 1.53 | 0.03 | 1.44 | 0.02 | 1.71 | 0.28 |
| MVMenket | 2.59 | 0.05 | 1.73 | 0.07 | 2.37 | 0.03 |
| Terzino | 1.87 | 0.04 | 1.22 | 0.02 | 1.16 | 0.03 |
| Tifi | 1.73 | 0.06 | 1.74 | 0.04 | 1.74 | 0.21 |

Table S3. Inhibitory activity against porcine pancreas α‑amylase [AIU/g] of the different wheat species and growing locations.

| Wheat species | Location | | | | | |
| --- | --- | --- | --- | --- | --- | --- |
| Common wheat | **Seligenstadt** | **±** | **Hohenheim** | **±** | **Eckartsweiher** | **±** |
| Event | 1594 | 170 | 2947 | 169 | 912 | 16 |
| Genius | 1535 | 16 | 580 | 40 | 870 | 16 |
| JB Asano | 1584 | 122 | 1130 | 119 | 1440 | 55 |
| Lear | 2622 | 265 | 637 | 100 | 730 | 102 |
| Mulan | 1385 | 114 | 1400 | 84 | 1298 | 81 |
| Tabasco | 2184 | 303 | 923 | 177 | 967 | 114 |
| Tobak | 2885 | 326 | 1251 | 151 | 1473 | 76 |
| Tommi | 683 | 111 | 820 | 128 | 867 | 175 |
| Spelt | | | | | | |
| Badengold | 1998 | 404 | 919 | 43 | 620 | 85 |
| Badenkrone | 320 | 24 | 380 | 13 | 376 | 19 |
| Badenstern | 345 | 7 | 1116 | 88 | 2829 | 80 |
| Filderstolz | 299 | 42 | 1290 | 207 | 682 | 68 |
| Franckenkorn | 370 | 24 | 1300 | 206 | 2684 | 537 |
| Oberkulmer Rotkorn | 350 | 12 | 1233 | 244 | 1883 | 52 |
| Schwabenkorn | 2873 | 274 | 1240 | 186 | 1346 | 188 |
| Zollernspelz | 2859 | 289 | 258 | 21 | 412 | 11 |
| Durum wheat | | | | | | |
| Auradur | 338 | 18 | 336 | 13 | 397 | 3 |
| W-05005/02 | 280 | 15 | 550 | 58 | 538 | 73 |
| Elsadur | 310 | 24 | 478 | 77 | 689 | 102 |
| Karur | 56 | 4 | 486 | 91 | 317 | 12 |
| Logidur | 153 | 17 | 134 | 18 | 188 | 6 |
| Lunadur | 76 | 5 | 795 | 42 | 776 | 64 |
| Lupidur | 187 | 11 | 187 | 12 | 215 | 7 |
| Wintergold | 314 | 11 | 140 | 13 | 214 | 15 |
| Emmer | | | | | | |
| CC1E-04058/01 | 77 | 7 | 1384 | 16 | 910 | 49 |
| CC1E-04059/01 | 84 | 3 | 880 | 147 | 826 | 90 |
| E-07087/02 | 76 | 4 | 73 | 4 | 77 | 3 |
| 9.105/06/01 | 59 | 4 | 311 | 61 | 528 | 48 |
| 9.121/05 Heuholzer Kolben | 44 | 4 | 264 | 12 | 2667 | 149 |
| Osiris | 74 | 5 | 212 | 27 | 3117 | 629 |
| Ramses | 74 | 3 | 67 | 3 | 77 | 3 |
| 9.131/05 Teutonia | 63 | 2 | 362 | 13 | 421 | 53 |

Table S4. Inhibitory activity against human saliva α‑amylase [AIU/g] of the different wheat species and growing locations.

| Wheat species | Location | | | | | |
| --- | --- | --- | --- | --- | --- | --- |
| Common wheat | **Seligenstadt** | **±** | **Hohenheim** | **±** | **Eckartsweiher** | **±** |
| Event | 1528 | 62 | 1206 | 36 | 1393 | 53 |
| Genius | 1691 | 19 | 2285 | 324 | 1468 | 112 |
| JB Asano | 1542 | 70 | 2649 | 309 | 3243 | 88 |
| Lear | 1477 | 113 | 3239 | 224 | 1483 | 90 |
| Mulan | 2961 | 140 | 2717 | 216 | 2903 | 232 |
| Tabasco | 3232 | 318 | 1384 | 178 | 1349 | 23 |
| Tobak | 3403 | 491 | 2190 | 191 | 3564 | 272 |
| Tommi | 897 | 73 | 1033 | 177 | 1304 | 108 |
| Spelt | | | | | | |
| Badengold | 1545 | 239 | 1546 | 84 | 1127 | 35 |
| Badenkrone | 1340 | 152 | 883 | 25 | 1282 | 155 |
| Badenstern | 908 | 14 | 1557 | 119 | 1266 | 31 |
| Filderstolz | 917 | 38 | 1691 | 118 | 1448 | 222 |
| Franckenkorn | 1712 | 12 | 1183 | 127 | 2803 | 258 |
| Oberkulmer Rotkorn | 1299 | 84 | 841 | 35 | 3154 | 130 |
| Schwabenkorn | 1614 | 83 | 1495 | 202 | 1551 | 47 |
| Zollernspelz | 3038 | 373 | 828 | 27 | 1446 | 148 |
| Durum wheat | | | | | | |
| Auradur | 841 | 29 | 784 | 69 | 852 | 14 |
| W-05005/02 | 793 | 28 | 733 | 82 | 800 | 35 |
| Elsadur | 780 | 41 | 733 | 90 | 824 | 10 |
| Karur | 811 | 27 | 623 | 46 | 422 | 64 |
| Logidur | 451 | 11 | 421 | 19 | 436 | 7 |
| Lunadur | 796 | 13 | 814 | 26 | 760 | 27 |
| Lupidur | 892 | 13 | 731 | 26 | 878 | 64 |
| Wintergold | 940 | 11 | 656 | 33 | 846 | 68 |
| Emmer | | | | | | |
| CC1E-04058/01 | 454 | 7 | 1324 | 115 | 899 | 42 |
| CC1E-04059/01 | 457 | 21 | 1461 | 207 | 891 | 13 |
| E-07087/02 | 446 | 21 | 179 | 7 | 448 | 13 |
| 9.105/06/01 | 619 | 66 | 675 | 23 | 766 | 26 |
| 9.121/05 Heuholzer Kolben | 727 | 75 | 692 | 37 | 714 | 36 |
| Osiris | 823 | 14 | 374 | 73 | 738 | 69 |
| Ramses | 439 | 13 | 170 | 4 | 453 | 9 |
| 9.131/05 Teutonia | 810 | 37 | 432 | 37 | 791 | 55 |
